# Effectiveness of noninvasive ventilation in acute heart failure edema secondary to acute coronary syndrome

**DOI:** 10.1186/2197-425X-3-S1-A170

**Published:** 2015-10-01

**Authors:** J Canovas, A Lopez, DO Torres, A Burruezo, L Capilla, A Carrillo

**Affiliations:** Hospital Morales Meseguer, ICU, Murcia Spain

## Introduction

The noninvasive ventilation (NIV) has been used successfully in acute respiratory failure (ARF) due to heart failure. However, patients with acute coronary syndrome have worse outcome than other ethologies of heart failure.

## Objetives

Primary objective: To analyze the effectiveness of NIV in patients with ARF due to acute heart failure due to acute coronary syndrome (AHF-ACS) or other causes (AHF-NACS). The secondary objective is to analyze the risk factors related to failure of NIV in these patients.

## Methods

Prospective study, for 16 years, in patients admitted to the ICU with ARF due to hear failure requiring treatment with NIV. The criteria for initiation of NIV were respiratory rate> 30, PaO 2 / FiO 2 < 250 or accessory muscles respiratory activity. The diagnosis of ACS was conducted by the European Society of Cardiology / American College of Cardiology criteria. Success of NIV was defined as avoiding intubation and be discharged alive plant. The results are expressed as mean ± standard deviation and absolute and relative frequencies. Comparisons between variables using Student T and Pearson Chi^2^. Multivariate logistic regression analysis.

## Results

During the study period, 1088 patients were admitted with AHF who required NIV, 453 (41.6%) with ACS. BiPAP mode was used in 993 patients (91.3%) and the rest CPAP. Do not intubate order in 258 patients (23.7%): Age at AHF-ACS group was 75 ± 9 and AHF-NACS 74 ± 10 (p = 0.235) and SAPS II: 43 ± 13 and 43 ± 12, respectively (p = 0.933). Respiratory parameters in patients and AHF AHF-ACS-NACS show: respiratory rate 37 ± 5 and 36 ± 5 (p: 0.187), pH: 7.35 ± 0.12 and 7.36 ± 0.12 (p:0.175) and PaO2/FiO 2: 127 ± 32 and 130 ± 32 (p = 0.101). LVEF was 32 ± 9 and 40 ± 13, respectively. Arterial hypotension at the time of initiation of NIV was 26.9% in AHF-ACS and 14.5% in AHF-NACS (p < 0.001). NIV success was obtained in 320 (70.6%) and 539 (84.9%) (p < 0.001) and hospital mortality in 342 (75.5%) and 560 (88.2%), respectively (p < 0.001). By multivariate analysis, predictors for NIV failure were: ACS (OR: 3.05; 95% -CI: 1.91-4.88; p < 0.001), SAPS II (OR: 1.05; 95% -CI: 1.03-1.07; p < 0.001), COPD (OR: 0.385 95% -CI: 0.19 to 0.78; p:0.008), respiratory rate 1 hour-NIV (OR: 1.09; 95% -CI: 1.04-1.14;p < 0.001), PaO2 / FiO2 1 hour-NIV (OR: 0.98; 95% -CI: 0.98 -0.99; p:0.008), NIV-related complication (OR: 4.98; 95% -CI: 3.04-8.16;p < 0.001), and SOFA maximum (OR: 1.54; 95% -CI: 1.43-1.67;p < 0.001).

## Conclusions

Patients with ACS as a cause of AHF have a greater number of failures of NIV, leading to a worse prognosis and causing increased mortality.

